# Stem cell-derived exosomes: roles in stromal remodeling, tumor progression, and cancer immunotherapy

**DOI:** 10.1186/s40880-015-0051-5

**Published:** 2015-09-14

**Authors:** Farah Fatima, Muhammad Nawaz

**Affiliations:** Department of Pathology and Forensic Medicine, Faculty of Medicine Ribeirao Preto, University of Sao Paulo, Av. Bandeirantes, 3900, Ribeirao Preto, Sao Paulo Brazil; Department of Rheumatology and Inflammation Research, University of Gothenburg, 480, 40530 Gothenburg, Sweden

**Keywords:** Stem cells, Exosomes, MicroRNAs, Fibroblasts, Stroma, Tumor microenvironment, Angiogenesis, Tissue repair, Transplantation, Immunotherapy

## Abstract

Stem cells are known to maintain stemness at least in part through secreted factors that promote stem-like phenotypes in resident cells. Accumulating evidence has clarified that stem cells release nano-vesicles, known as exosomes, which may serve as mediators of cell-to-cell communication and may potentially transmit stem cell phenotypes to recipient cells, facilitating stem cell maintenance, differentiation, self-renewal, and repair. It has become apparent that stem cell-derived exosomes mediate interactions among stromal elements, promote genetic instability in recipient cells, and induce malignant transformation. This review will therefore discuss the potential of stem cell-derived exosomes in the context of stromal remodeling and their ability to generate cancer-initiating cells in a tumor niche by inducing morphologic and functional differentiation of fibroblasts into tumor-initiating fibroblasts. In addition, the immunosuppressive potential of stem cell-derived exosomes in cancer immunotherapy and their prospective applications in cell-free therapies in future translational medicine is discussed.

## Introduction

Cancer stem cells (CSCs) are tumor cells with substantial potential for self-renewal, clonal tumor initiation, long-term repopulation, and phenotypic plasticity preservation [1, 2]. A number of cell surface markers, such as CD34, CD133, CD24, CD44, CD166, and epithelial cell adhesion molecule (EpCAM), are used to identify and enrich CSCs from several types of cancer [3–6]. Although the origin of CSCs and the prediction of their biological activity are controversial subjects, they have been explained using a CSC model (also known as a hierarchical model). This model posits that a defined subset of biologically distinct cells is solely responsible for initiating malignancy [7, 8] and that the tumor population is hierarchically arranged within the tumor niche [9]. Subsequently, the tumor-initiating activity can be enriched by sorting cells on the basis of intrinsic characteristics and may be prospectively isolated based on a specific cell surface phenotype [7].

In contrast, the stochastic model of cancer posits that all of the cells within a cancer have equal potential to act as cancer-initiating cells (CICs) that propagate the cancer [10–12]. Tumor-initiating activity cannot be improved by sorting cells based on intrinsic characteristics and cannot be prospectively determined based on the environment in which the cells reside. This model assumes that the activities of CICs are governed by re-entry into the cell cycle, which is in fact a low-probability stochastic event, making it impractical to realistically identify the tumor-initiating subset.

The last decade has witnessed remarkable progress in stem cell-based therapies with promising clinical applications (reviewed in [13]). Recent trends in translational medicine are reliant on the application of stem cells, largely because of stem cells’ potential in tissue regeneration and defect repair. The potential use of stem cells for both cellular and non-cellular therapies could offer a promising opportunity to treat tumors and immune diseases for which existing therapeutic strategies pose potential challenges.

Of prime importance are mesenchymal stem cells (MSCs), which represent a heterogeneous pluripotent population of plastic-adherent cells that exhibit a fibroblast-like morphology and form distinct colonies when seeded at clonal densities [14]. MSCs are commonly derived from bone marrow (BM), adipose tissue, nervous tissue, amniotic fluid, the umbilical cord, the placenta, menstrual blood, and even dental pulp [15], with differences in morphology, differentiation, proliferation and self-renewal ability, and multilineage differentiation potential [16]. Remarkably, the differentiation capacity of MSCs may have potential clinical implications for the treatment of various diseases, as these cells could be activated and recruited to sites of tissue damage, where they could induce regenerative programs for repairing defects. Although these cells’ differentiation potential is less than that of embryonic stem (ES) cells and induced pluripotent stem (iPS) cells, MSCs hold greater potential for clinical applications. Intriguingly, the most prominent therapeutic effect of MSCs is based on their immunoregulatory functions [15].

The biological effects and immunoregulatory functions of MSCs depend largely on secreted factors that potentially stimulate tissue-intrinsic progenitor cell programs and may promote the differentiation and tissue-reparative properties of MSCs [15, 17]. Importantly, the secreted factors are critical in mediating crosstalk between the elements of the local stroma and stem cells, whereby the cells may assertively orchestrate themselves in a given tumor niche and may exchange cytoplasmic and genetic material. The fusion and exchange of biological material are vital to cancer development and could therefore exhibit several features of CSCs [18]. In this regard, the exchange of oncogenic material, including stem cell signatures, through secretory vesicles (exosomes) from stem cell hierarchies could represent several features of the originating cells. Therefore, we speculate that exosomes released from CSCs may reflect CSC features and that the morphologic and functional relevance of exosomes solely specific to CICs may potentially contribute to resolving the ambiguities in cancer-initiating activities and enrichment, which are hotly debated issues in the stochastic cancer model and the CSC model. However, further studies will be needed to link exosomes and the two cancer-initiating models to explain exosome-based cancer-initiating activities and enrichment.

Exosomes are nano-sized (40–200 nm) vesicles secreted by many types of cells and can be detected in various body fluids [19, 20]. The isolation and purification of exosomes can be achieved through a variety of conventional and high-throughput technologies, depending largely on the source of exosomes and the relative choice of downstream analysis (reviewed comprehensively in [20]). Exosomes carry enormous populations of bioactive molecules, such as mRNAs, microRNAs (miRNAs), and long non-coding RNAs (lncRNAs) [21–24]; genomic DNA, cDNA, and mitochondrial DNA (mtDNA) [25–31]; and proteins and lipids [32–36]. Exosomes may transfer unique patterns of their contents to neighboring cells and may therefore induce the phenotypic modifications in recipient cells, subsequently modulating the microenvironment [37, 38]. Interestingly, the molecular signatures of exosomes are specific to each tissue type, which makes exosomes ideal for clinical applications [20].

Accumulating data have clarified that exosomes may play vital roles in the pathophysiology of various diseases [39–48], with well-known effects on the development and progression of cancer [20, 49, 50]. Most often, exosomes promote cancer by suppressing immune surveillance mechanisms and fostering the development of drug resistance [51, 52], thus offering potential clinical applications in tumor targeting and vaccine development. Strikingly, immunosuppressive exosomes of tumor origin can maintain tumor growth through stromal remodeling, pro-angiogenic growth factor expression, intercellular genetic exchange, and chemoresistance [53]. Therefore, exosomes secreted by immunoreactive stem cells may represent a versatile resource for future cancer immunotherapy.

## Biogenesis of exosomes

The biogenesis of exosomes follows the endocytic-exocytic pathway, which involves various cellular compartments, such as early and late endosomes, lysosomes, and multivesicular bodies (MVBs). The precise mechanisms of biogenesis are not fully understood, but it is well known that sorting of exosomal cargo is a tightly regulated process that occurs under the influence of different sorting machineries (for the detailed mechanisms, see [20]). Once exosomes are sorted, they are released outside the cell following the fusion of MVBs with the plasma membrane.

**Fig. 1:**
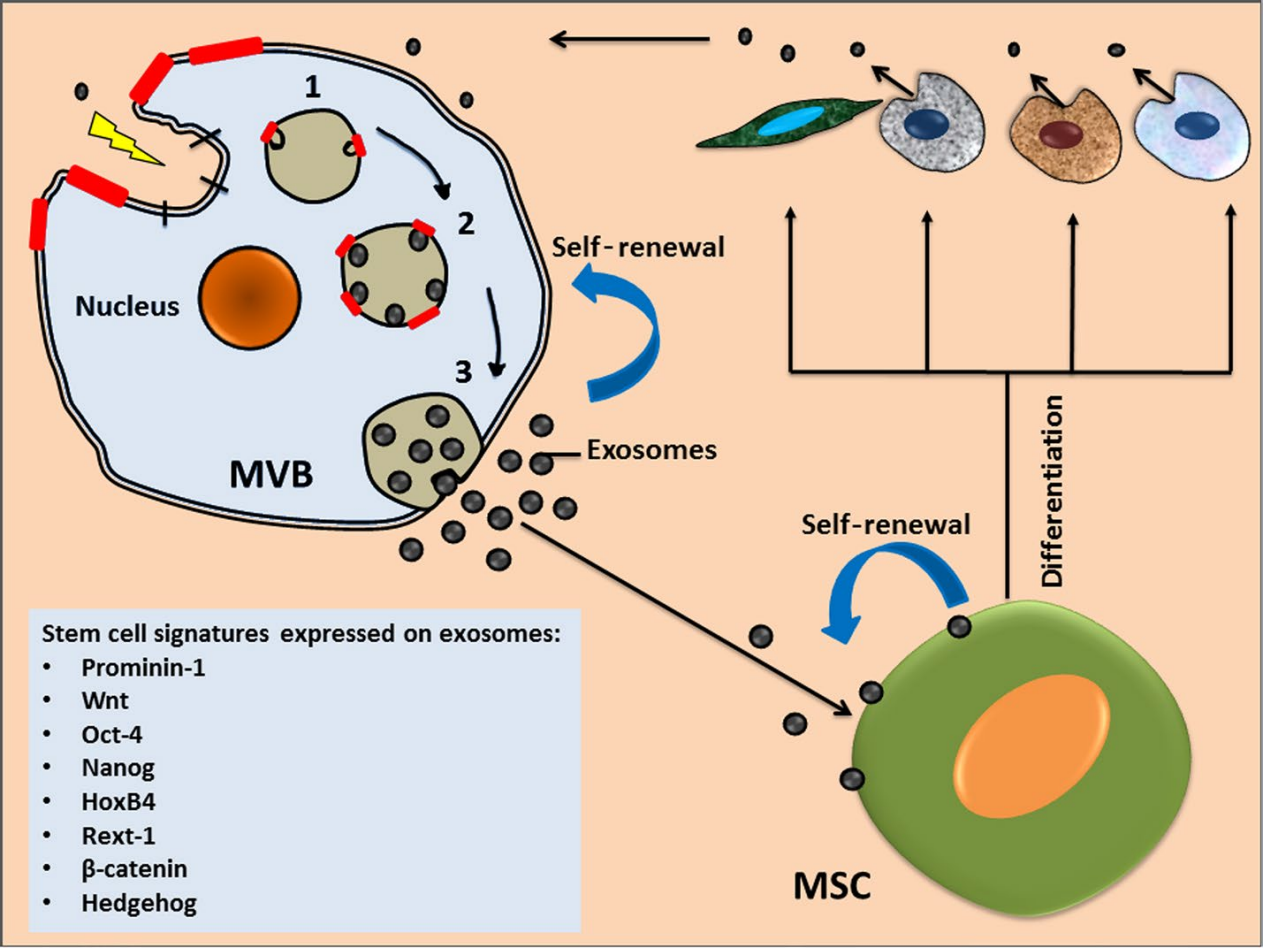
Biogenesis of exosomes and their role in maintaining stemness: exosome formation follows the endocytic-exocytic pathway, which is initiated upon receiving extrinsic or intrinsic signals in a context-dependent manner, i.e., in response to either a disease signal or a reparative signal related to tissue injury. The regions of plasma membrane enriched in lipid-raft microdomains (*red bars*) are inveginated, resulting in the formation of early endosomes and their maturation into late endosomes (steps 1–3), with a series of changes occurring under the influence of multicomponent sorting machinery [20]. Multivesicular bodies (MVBs) fuse with the plasma membrane and secrete exosomes outwardly; these exosomes may interact with resident cells and may facilitate stemness, i.e., self-renewal, clonal expansion, differentiation, and repair. MSCs, mesenchymal stem cells

The formation and secretion of exosomes by biologically active cells are arguably context-dependent, i.e., arising in response to repair processes or tumor progression. MSC-derived exosomes are originated from endocytosed lipid-raft microdomains [54], whereas the endocytic-exocytic pathway may play a role in stem cell differentiation through the release of membrane vesicles containing stem cell markers, such as prominin-1/CD133 [55]. Upon their release from stem cells, secreted exosomes participate in the maintenance of stem cell hierarchies (Fig. 1). Notably, the knowledge about the mechanisms of the biogenesis and origin of exosomes from stem cells could enhance our understanding of exosomes’ functional relevance and could aid in the development of strategies for engineering exosomes for prospective therapeutic applications.

## Role of stem cell-derived exosomes in maintaining stemness and regeneration

Secreted exosomes exhibit fundamental paracrine mechanisms that mediate cell-to-cell communication through direct receptor-mediated stimulation of target cells and the horizontal transfer of genetic material [22, 38, 56]. It has been proposed that the exosome-mediated exchange of genetic information could be an integral element of the continuum model of stem cell biology, in which the differentiation decision of stem cells is conditioned by cell cycle transit and possibly by environmental stimuli [57]. In this context, as paracrine factors, stem cell-derived exosomes might induce phenotypic changes in recipient cells through genetic exchange and might generate a functional link between stem cells and resident tissue under various physiologic and pathologic conditions.

Intriguingly, exosomal mRNA transcripts for pluripotent transcription factors such as Nanog, Oct-4, HoxB4, Rex-1, and the stem cell-specific Wnt-3 protein, all expressed in stem cell-derived exosomes, may represent several features of stemness, such as maintenance of self-renewal properties and expansion of progenitor cells [21]. Several studies have indicated that stem cell effectors such as WNT [58, 59], Hedgehog [60], and β-catenin [61], which are expressed on stem cell-derived exosomes, may play a potential role in maintaining stemness (Fig. 1). Importantly, several other reports have clearly indicated that stem cell signatures expressed on exosomes play a vital role in maintaining stem cell characteristics, such as differentiation, self-renewal, and maturation [55, 62–65].

In fact, stem cell-derived exosomes may represent several features of their cells of origin and may play a role in gain-of-function properties, genetic instability, and consequent malignant transformation as well as the induction of reparative programs within injured tissues.

## Induction of regenerative phenotypes

Exosomes released from MSCs are a newly discovered source of regeneration in injured tissues, participating in exosome-mediated transfer of regenerative molecules to target cells in a paracrine fashion. For example, stem cell-derived exosomes participate in the suppression of inflammatory responses through the inhibition of hyperproliferative pathways, thus restoring bioenergetics to promote cell survival [66–69]. Moreover, these exosomes may have the potential to reduce infract size and ameliorate heart functions through promoting neovascularization [70–73]. MSC-derived exosomes appear to have multi-pluripotent effects on neurovascular plasticity in rats during traumatic brain injury by promoting endogenous angiogenesis and neurogenesis accompanied by reduced neuro-inflammation [74, 75]. The anti-inflammatory and protective effects of MSC-derived exosomes were also observed during sciatic nerve regeneration in rats that received peripheral nerve cell therapy [76].

Stem cell-derived exosomes may play a role in accelerating the morphologic and functional recovery of the liver through exosome-mediated horizontal transfer of specific mRNA subsets [77], activation of proliferative and regenerative responses [78], reduction in hepatic inflammation, and inhibition of the epithelial-mesenchymal transition in the fibrotic liver [79]. Similarly, acute and chronic lung injuries may be recovered through the secretion of exosomes by MSCs, conferring stem cell-like phenotypes on injured cells to promote self-regenerative programs [80, 81]. Interestingly, the protective effects of MSC-derived exosomes are comparable to those of MSCs, as could be observed in renal recovery [82–85] and wound and bone healing [86–88], indicating that exosomes are functional extensions of MSCs.

## Anti-apoptotic effect of MSC-derived exosomes and tissue regeneration

Exosomes released from MSCs may potentially stimulate cell survival by inducing anti-apoptotic effects in injured cells and by promoting cell proliferation, as has been well explained for cardiomyocytes [73, 89] and injured renal tubular epithelial cells [90–93]. In fact, exosomes transfer anti-apoptotic miRNAs from stem cells to injured tissues and may contribute to cell survival by transcriptionally repressing the expression of apoptotic genes and activating cell survival signaling. Exosomes released from MSCs and iPS cells could deliver cardioprotective miRNAs to cardiomyocytes in vitro, thereby producing cytoprotective effects and subsequently ameliorating heart function [94, 95]. In addition, exosome-mediated transfer of miRNAs to cardiac progenitor cells may play a role in promoting repair programs in the heart [96, 97].

Exosome-mediated transfer of miRNAs from MSCs promotes neural plasticity and functional recovery after treatment of stroke in the rat brain [98] and may also increase neuronal differentiation of neural progenitor cells [99]. Anti-apoptotic effects of MSC-derived exosomes could also be observed during rat pheochromocytoma protection through apoptotic regulation, i.e., through down-regulation of the expression of Bax, accompanied by reduced cleavage of caspase-3 and up-regulation of Bcl-2 [100]. These findings imply that transplantation of MSC-derived exosomes has enormous potential to protect against cerebral injury and possibly other neuronal diseases.

## Role of stem cell-derived exosomes in tumor progression and inhibition

Several lines of evidence indicate the prospective roles of MSCs and their secreted exosomes in initiating tumor phenotypes. For example, in human renal cell carcinoma, a subset of tumor-initiating cells expressing the MSC marker CD105 release exosomes that promote tumor growth [101, 102], probably through enhanced expression of genes associated with cell migration, matrix remodeling, and angiogenesis [102]. Exosomes from adipose-derived MSCs (AT-MSCs) are enriched in distinct patterns of RNAs that may potentially induce adipogenesis, angiogenesis, apoptosis, and proteolysis in recipient cells [103]. Exosomes from gastric cancer-derived MSCs may deliver miRNAs to human gastric cancer cells and promote their proliferation and migration [104]. Recently, it was shown that glioma-associated stem cells produce substantial amounts of exosomes that exhibit glioma stem cell features and that are capable of sustaining the malignant properties of both glioma cells and glioma stem cells, mainly through the release of exosomes [105].

Moreover, exosomes from BM-derived MSCs (BM-MSCs) transport tumor regulatory miRNAs, anti-apoptotic proteins, and metabolites that promote breast tumor growth [106]. Exosomes from BM-MSCs express higher levels of oncogenic proteins, cytokines, and adhesion molecules, which can be transferred to multiple myeloma cells, subsequently modulating tumor growth in vivo [107].

**Fig. 2:**
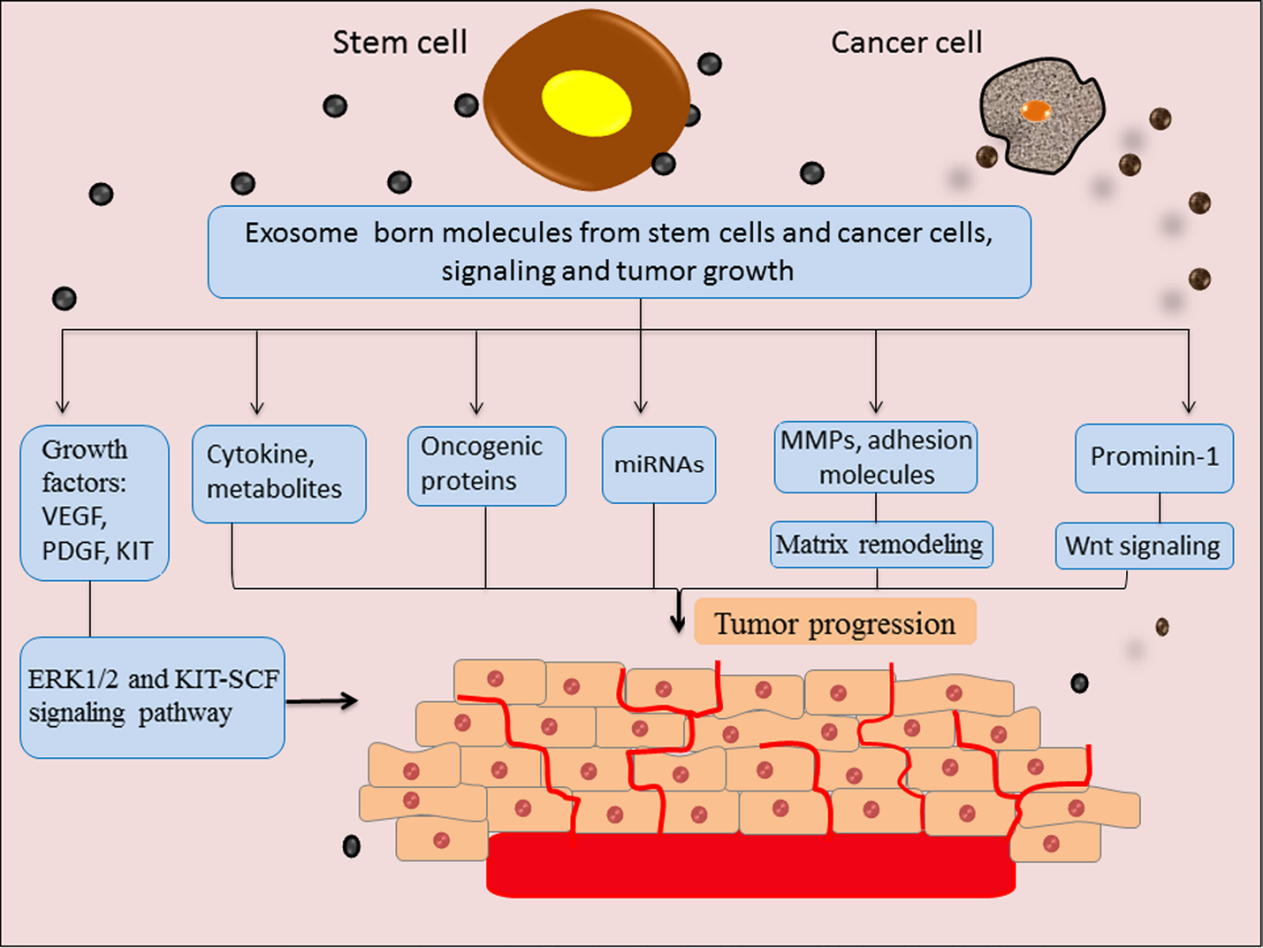
Stem cell-derived exosomes and tumor progression: exosomes derived from stem cells or cancer cells express a variety of molecules, comprising oncogenic microRNAs (miRNAs) and proteins, growth factors, and adhesion molecules that exhibit pro-metastatic and pro-angiogenic activities through activating signaling pathways. *VEGF* vascular endothelial growth factor, *PDGF* platelet-derived growth factor, *KIT* mast/stem cell growth factor receptor, *MMPs* matrix metalloproteinases, *ERK1*/*2* extracellular signal-regulated kinase 1/2, *SCF* stem cell factor

Lately, it has become apparent that stem cell-derived exosomes may contribute to tumor progression due to these exosomes’ ability to activate signaling pathways by expressing exosome-borne growth factors. For instance, exosomes from BM-MSCs appear to enhance the expression of vascular endothelial growth factor (VEGF) through the activation of the extracellular signal-regulated kinase 1/2 (ERK1/2) signaling pathway to promote tumor growth [108]. Exosomes from mast cells express and shuttle mast/stem cell growth factor receptor Kit (KIT), a member of the tyrosine kinase family of growth receptors which promotes tumor growth in recipient lung adenocarcinoma cells by activating the KIT-stem cell factor (SCF) signaling pathway [109]. Given that the Wnt signaling pathway is central to stem cell biology [110, 111], exosomes expressing the stem cell marker prominin-1 may play a potential role in Wnt signaling and pro-metastatic activity in melanoma cells [112]. Taken together, these observations indicate that stem cell-derived exosomes potentially express tumor-promoting molecules, such as miRNAs, oncogenic proteins, growth factors, and adhesion molecules, that engage in pro-metastatic and pro-angiogenic activities by activating signaling pathways (Fig. 2).

Intriguingly, MSC-derived exosomes not only have pro-tumor potencies but also can exert negative effects on tumor growth, depending on the conditions, the tumor type, and the stage of development [113] as well as the expression of tumor suppressor molecules. For example, exosomes from BM-MSCs act as negative regulators of the cell cycle and exert inhibitory effects on tumor growth [114]. Moreover, exosomes from BM-MSCs can transfer miRNAs from the BM and promote dormancy in metastatic breast cancer [115]. Breast cancer growth can also be inhibited by MSC-derived exosomes through miRNA-mediated VEGF suppression [116]. Similarly, exosome-mediated delivery of selective miRNAs from human liver stem cells may inhibit hepatoma growth [117]. Katakowski et al. [118] have shown that intra-tumoral injection of MSC-derived exosomes expressing *miR*-*146* could effectively inhibit glioma xenograft growth. MSC-derived exosomes are capable of incorporating and delivering paclitaxel, which can inhibit tumor growth [119], indicating that stem cell-derived exosomes possess the potential for drug delivery to cancer cells.

**Fig. 3:**
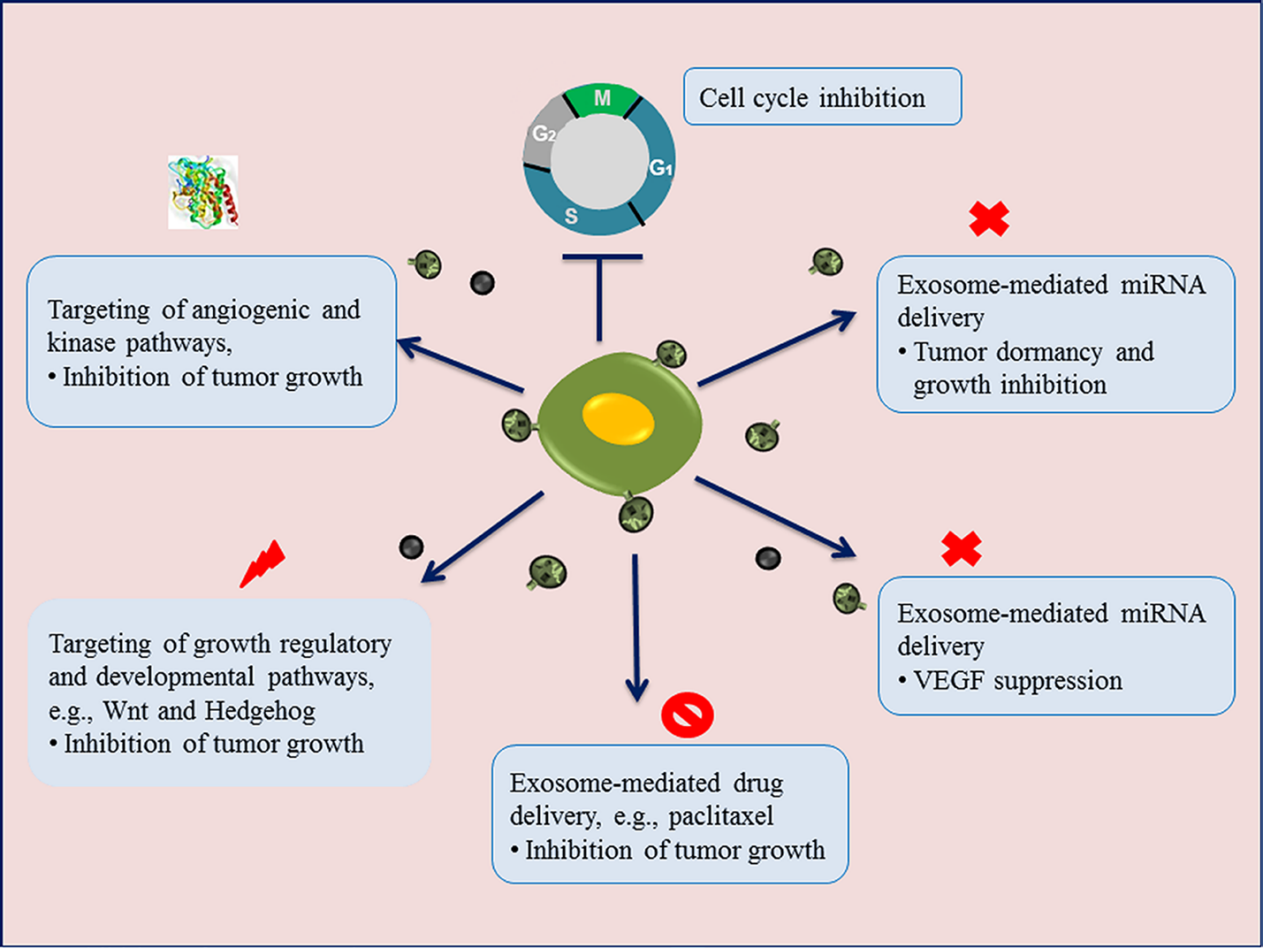
Stem cell-derived exosomes and tumor inhibition: exosomes express and deliver antitumor molecules that exhibit tumor suppressor activities in recipient cells and that potentially inhibit tumor growth by targeting angiogenic, growth-regulatory, and other signaling pathways

Exosome-mediated delivery of tumor suppressor miRNAs and targeting of growth-regulatory pathways, such as the Wnt and Hedgehog pathways, as well as angiogenic pathways, such as the VEGF and kinase pathways, could be novel strategies to monitor tumor growth (Fig. 3). For example, the potent signaling axis miR-140/SOX2/SOX9, which regulates differentiation, stemness, and migration, could be targeted to obstruct tumor progression [120]. Similarly, exosomes from MSCs could be effective in inhibiting bladder tumor cell growth by down-regulating the phosphorylation of Akt kinase [121], whereas exosome-mediated targeting of the VEGF pathway could offer a novel strategy to inhibit tumor growth by inhibiting angiogenesis [116]. However, it remains an open technical challenge to monitor the complex stromal network and to target these pathways within the dynamic tumor microenvironment.

## Mechanisms

### Establishment of pre-metastatic niche

The principal properties of CSCs are maintained by niches that are anatomically distinct regions within the tumor microenvironment [122]. Intriguingly, the pre-metastatic niche may play a role in dormancy, relapse, and the development of metastasis. It has been hypothesized that exosomes may act as metastasomes, helping to establish secondary lesions by transmission of the metastatic phenotypes to the target organ via an exosome-borne tumor RNA signature [123]. Given that the construction of a pre-metastatic niche is an essential early step for CICs to survive and evolve [124], it could be speculated that stem cells may contribute to the construction of the tumor-initiating niche at least in part by secreting exosomes. This concept may be further supported by observations that the interactions between endothelial cells and CSCs induce phenotypic changes in MSCs and promote the formation of a lung pre-metastatic niche through the release of exosomes [101].

Exosomes released from a subset of CICs could induce an angiogenic phenotype in endothelial cells and could promote the formation of a pre-metastatic niche [101, 102]. In fact, angiogenesis is one of the underlying mechanisms that shapes the tumor niche and is propagated by pro-angiogenic growth factors such as VEGF and platelet-derived growth factor (PDGF) [125]. In this regard, stem cell-derived exosomes appear to exert their pro-angiogenic effects by promoting enhanced expression of VEGF in tumor cells [108]. In response to hypoxia, MSCs release an elevated level of exosomes, which may promote endothelial cell growth in vitro [126] and thus may potentially induce angiogenesis [127]. Exosomes released from AT-MSCs interact with endothelial cells and may transport angiogenic factors and subsequently promote angiogenic activity in a tumor niche [128]. It has been shown that exosomes released from adipose stromal cells (ASCs) are responsible for ASC-induced angiogenesis, whereas PDGF triggers an angiogenic effect by stimulating ASCs to release more exosomes [129], which may play a role in shaping a permissive tumor microenvironment.

### Exosome-mediated crosstalk among stromal elements

The general involvement of exosomes in intercellular communication suggests that they may contribute to the exchange of biological information within stem cell hierarchies, and thus, cancer stem-like cells may transmit signals to their stroma by secreting exosomes. The exosome-mediated dynamic crosstalk within stromal elements may mobilize and re-localize the oncogenic factors that may shape the tumor environment. This speculation is based on the fact that carcinogenesis involves the re-localization of cancer-associated fibroblasts (CAFs) to the tumor site, sustaining metastasis [130].

Several studies have demonstrated that stromal cell-derived exosomes can also interact with cancer cells and can exchange oncogenic signatures present in tumor-associated stroma. For example, intercellular communication mediated by fibroblast-derived exosomes promotes cancer cell motility via autocrine Wnt-planar cell polarity (PCP) signaling to drive invasive activities [58]. Considering the mechanism of cell-to-cell communication, Wnt3a could be exported to neighboring cells via exosomes that may modulate population equilibrium in the tumor niche [131]. Such features are expected to be established very early during tumorigenesis, whereas the prolonged communication could sustain and aggravate tumor growth. It may be anticipated that the tumor microenvironment may also host multipotent stem cells that, by themselves, are non-tumorigenic but can support the biological activity of CICs through the release of exosomes.

### Exosome-mediated fibroblastic differentiation

The process of CSC plasticity, in which cancer cells present the dynamic ability to switch from a non-CSC state to a CSC state, can be modulated by secreted factors and cellular interactions within a tumor niche [132]. In this regard, exosome-mediated interactions as well as fibroblastic differentiation can implement switching of fibroblastic phenotypes into CAFs. In fact, tumor cell-derived exosomes may reshape the tumor niche through fibroblastic differentiation of MSCs. This concept implies that through the release of exosomes, tumor cells can “educate” MSCs towards CIC phenotypes arising within the microenvironment [133].

In fact, exosomes from cancer cells are capable of inducing ASCs to acquire the characteristics of tumor-supporting myofibroblasts [134] and may promote phenotypic differentiation of MSCs into CAFs [135]. Chowdhury et al. [136] have reported a consistent phenotypic switch that operates via prostate cancer-derived exosomes, which promote the differentiation of MSCs into pro-angiogenic and pro-invasive myofibroblasts. Similarly, prostate cancer-derived exosomes induce phenotypic transformation of ASCs in prostate cancer patients to promote prostate cancer-like neoplastic lesions [137]. Shimoda et al. [138] have proposed that activated CAFs influence tumor progression through the secretion of metalloproteinase-enriched exosomes that promote cell motility and activate RhoA and Notch signaling in cancer cells. Surprisingly, exosomes from CAFs play a role in tumor recurrence and chemoresistance through promoting the clonogenicity of CSCs, which are inherently resistant to cell death [139].

### Potential of stem cell-derived exosomes in cancer immunotherapy

Due largely to their immunogenicity, these tiny immunosomes have emerged as potential vectors for cancer immunotherapy. Exploiting the immunogenicity of exosomes, they could be implemented in clinical settings and in the development of vaccination [140–143]. In particular, exosomes of tumor origin could be engineered to deliver a potent immunogen with an ability to induce effective immune responses in recipient cells [144], representing a novel type of tumor vaccine.

Although a large amount of data have shown the potential of exosomes in regulating immune mechanisms [145–147], the clinical information about stem cell-derived immunoregulatory exosomes is limited. However, emerging evidence has shown that MSCs are remarkably capable of secreting immunologically active exosomes, which may serve as a potent platform for stem cell-based cancer immunotherapy. Initial studies have indicated that exosomes secreted by human breast tumor cells could inhibit the differentiation of human monocytes in vitro [148], suggesting that tumor-derived exosome-mediated stimulation of murine myeloid precursors and induction of interleukin (IL)-6 play a critical role in immune modulation and inhibition of differentiation. These findings imply that stimulation of precursors by tumor-derived exosomes could open an opportunity to use exosomes in cancer immunotherapy.

A recent study showed that hematopoietic progenitor cell antigen CD34-containing exosomes from acute myeloid leukemia (AML) blasts appear to be immunoreactive [149]. Surprisingly, upon co-incubation with natural killer (NK) cells, captured blast-derived exosomes efficiently down-regulate the expression of surface NK group 2, member D (NKG2D). This result indicates that blast-derived exosomes can retain the ability to mediate immune suppression. Moreover, immunocaptured blast-derived exosomes might be useful biomarkers for AML during the course of immunotherapy as a measure of disease progression as well as of the response to therapy.

Regulation of cytokine expression and immune cells by stem cell-secreted exosomes may also play a role in modulating inflammatory responses. For example, MSC-derived exosomes could induce higher expression of anti-inflammatory IL-10 and tumor growth factor-β1 (TGF-β1) transcripts and reduce the levels of pro-inflammatory IL-1B, IL-6, tumor necrotic factor α (TNFα), and IL-12P40 transcripts [150], suggesting the use of these exosomes in immunotherapies for inflammatory diseases. MSC-derived exosomes exert inhibitory effects on the differentiation and activation of T cells, followed by reduced T cell proliferation and interferon-γ (IFN-γ) release, suggesting that MSC-derived exosomes are therapeutic agents for the treatment of inflammation-related diseases [151]. Exosomes from MSCs are strictly immunostimulatory in their biological activities, which can activate autoreactive B and T cells in non-obese diabetic mice [152]. In fact, immunization with exosomes may promote the expansion of transferred diabetogenic T cells and accelerate the effector T-cell-mediated destruction of islets, suggesting that stem cell-derived exosomes are autoantigen carriers, serving as prospective adjuvant therapy [152].

**Fig. 4:**
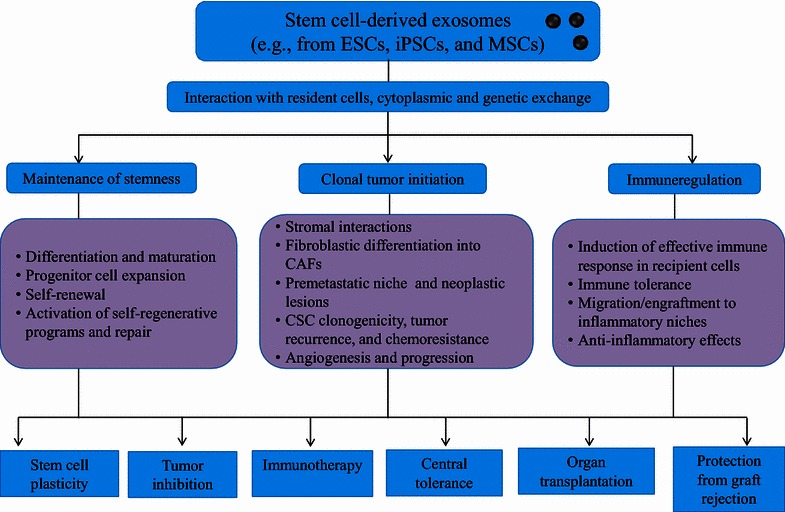
Stem cell-derived exosomes, their role in maintenance of stemness and tumor propagation, and their clinical potential in immunotherapy and organ transplantation. *ESCs* embryonic stem cells, *iPSCs* induced pluripotent stem cells, *CAFs* cancer-associated fibroblasts, *CSC* cancer stem cells

In summary, the multipotential aspects of stem cell-derived exosomes in maintaining stem cell features and regulation of immune programs in recipient tissues could be a future clinical consideration for disease monitoring (Fig. 4). Although augmenting data support the opinion that exosomes could be a promising therapeutic resource for the treatment of immune diseases [43, 153], translating this technology into stem cell-derived exosome-mediated cancer immunotherapy that offers minimized toxic effects and safety risks will need further studies.

## Concluding remarks and future perspective

Despite improvement both in the surgical procedures for organ transplantation and in cell-based therapies over the last decade, current methods present potential complications (for example, an increased risk of infection, toxicity, and graft rejection). In this context, exosomes may offer a nontoxic framework of protective molecules during transplantation, as they are capable of inducing endogenous regenerative programs in damaged tissues.

Compared with traditional stem cell therapies, exosome-based cell-free therapy may improve patients’ outcomes considerably, reducing the complications of cell-based therapy. However, steering traditional stem cell-based therapy toward exosome therapy will need further studies. Intriguingly, the combination of exosome-based therapies with existing traditional approaches could improve the therapeutic benefits. The intriguing advantages of stem cell-derived exosomes in therapy are largely due to exosomes’ ability to stimulate endogenous repair processes within the injured tissue and their efficient regulation of immune tolerance. Therefore, the administration of exosomes for prospective therapeutic applications may help to minimize the potential safety risks associated with cellular therapy or transplantation surgery, preventing graft rejection. Manipulating the expression of exosomal contents related to angiogenesis, growth, proliferation, and self-renewal could potentially improve blood flow recovery, may maintain tissue growth, and thus could ultimately ameliorate organ function.

Strikingly, haploidentical MSC transplantation could efficiently postpone the rejection of mismatched skin grafts in patients with severe treatment-resistant acute graft-versus-host disease (GVHD) [154], whereas immunosuppressive exosomes could potentially prevent graft rejection [155]. These findings raise the possibility that immunosuppressive exosomes from MSCs could be a promising therapeutic resource for organ transplantation, preventing graft rejection with great efficacy. An example could be seen when an infusion of MSC-derived exosomes significantly enhances survival and delays the rejection of an allogenic skin graft, as observed in mice, with a corresponding increase in regulatory T cells [150]. Thus, the application of MSC-derived exosomes could be a novel therapeutic platform for future clinical consideration due to the fact that MSC-derived exosomes are well tolerated in patients during the treatment of GVHD [156]. Moreover, MSC-derived exosome treatment could efficiently reduce the pro-inflammatory cytokine response in patients’ peripheral blood mononuclear cells in vitro, and the clinical symptoms of GVHD could be improved significantly shortly after the start of MSC-derived exosome therapy [156]. Importantly, the exosome contents before and after BM-MSC treatment may potentially modulate the immune response [157], suggesting that the exosomal contents of MSC products will be a consideration when used in the clinic in the future.

Despite that stem cell-derived exosomes have been employed in clinical practice for tissue repair and organ transplantation, their application in treating cancers seems challenging. The poor survival of cancer patients, which is mainly associated with the late stages of cancer, a lack of early diagnosis, recurrence, and resistance to existing therapies, poses a potential challenge for improving the long-term survival of patients and reducing the risks of mortality. In this regard, exosomes could serve as a promising resource for early diagnosis, enabling timely monitoring of the disease [20], as well as for developing novel cancer therapies alternative to existing therapies [158]. Cancer immunotherapy principally relies on evoking the host immune system to fight against cancer cells, whereas the immunosuppressive tumor microenvironment is considered a major barrier to the effectiveness of cancer immunotherapies [159]. Given that exosomes play a vital role in central tolerance, efficiently stimulating the immune system and potentially reshaping the immunosuppressive microenvironment [160], this potential could be exploited for the use of stem cell-derived exosomes in cancer immunotherapy, serving as a potent resource of antitumor agents. It is even more likely that exosomes will also serve as a measure of disease progression as well as the response to therapy during the course of immunotherapy.

It is well appreciated that the human T-cell response can be modified using viral and non-viral vectors to promote the selective targeting of cancer cells by introducing exogenous T-cell receptors (TCRs) or chimeric antigen receptors (CARs) [161]. In this regard, using exosomes as vectors for introducing TCRs and/or CARs that specifically target cancer cells may raise the novel possibility of using exosomes for cancer immunotherapy. However, to validate this possibility, further studies will be needed.

Because they are free of potential safety risks, are non-toxic, and do not differentiate into unrequired populations, in contrast to CSCs, exosomes could be a promising platform for cancer therapies. Moreover, precise understanding of the involvement of exosomes in CSC biology and their prospective potential in the prediction and enrichment of cancer-initiating activities may present novel opportunities for developing effective therapies against cancer. Importantly, the use of exosomes, and particularly those derived from MSCs, do not raise potential ethical and legal issues, in contrast to other types of stem cells, and especially human embryonic stem cells (ESCs). Of particular note, the cost of exosome engineering and manipulation would be significantly less, which will be an important clinical consideration.

Despite the enormous potential of exosomes in future clinical settings, strategies will need to be developed for the precise and adoptive manipulation of exosomes for effective usage in local transplantation, systemic infusion, and engineering of their clinical benefits for cancer therapy. Moreover, whether the dose-dependent effects of exosomes in cancer model experiments are promising must be determined because increasing the dose of exosomes may produce unwanted phenotypes and may limit the accessibility of their application to a smaller number of patients. A potential challenge in the field exists due largely to the limitations of standardizing the existing technologies for the isolation, characterization, and engineering of exosomes for their prospective therapeutic use. Therefore, a combination of different high-throughput approaches and precisely optimized methods could overcome the potential limitations related to exosomal detection, characterization, and manipulation [20], which may be implicated in biomarker development and possibly in the clinical use of stem cell-derived exosomes in future cancer therapies [162].
